# Plant Growth-Promoting Bacteria of Soil: Designing of Consortia Beneficial for Crop Production

**DOI:** 10.3390/microorganisms11122864

**Published:** 2023-11-26

**Authors:** Anna M. Timofeeva, Maria R. Galyamova, Sergey E. Sedykh

**Affiliations:** 1 SB RAS Institute of Chemical Biology and Fundamental Medicine, 630090 Novosibirsk, Russia; anna.m.timofeeva@gmail.com; 2 Faculty of Natural Sciences, Novosibirsk State University, 630090 Novosibirsk, Russia; mgalyamova@gmail.com

**Keywords:** rhizobacteria, microbe–plant interaction, PGPB, microbial communities, synthetic microbial communities, bacterial interactions, consortium modeling, symbiosis, consortium, designing synthetic microbial communities

## Abstract

Plant growth-promoting bacteria are commonly used in agriculture, particularly for seed inoculation. Multispecies consortia are believed to be the most promising form of these bacteria. However, designing and modeling bacterial consortia to achieve desired phenotypic outcomes in plants is challenging. This review aims to address this challenge by exploring key antimicrobial interactions. Special attention is given to approaches for developing soil plant growth-promoting bacteria consortia. Additionally, advanced omics-based methods are analyzed that allow soil microbiomes to be characterized, providing an understanding of the molecular and functional aspects of these microbial communities. A comprehensive discussion explores the utilization of bacterial preparations in biofertilizers for agricultural applications, focusing on the intricate design of synthetic bacterial consortia with these preparations. Overall, the review provides valuable insights and strategies for intentionally designing bacterial consortia to enhance plant growth and development.

## 1. Introduction

Microbial communities associated with plants are called the plant microbiome [1]. They are critical for plant health and adaptation to environmental factors [2]. The interactions between plants and soil microbial communities can be very complex. When the soil microbiome is formed, plants select the microbial partners that could improve their growth, development, and productivity. The soil microbiota is supplied with nutrients secreted by plants as root exudates [3].

To date, only a few individual effects mutually exerted by plants and microbes have been well characterized, for example, nitrogen fixation by rhizobia. Developing novel strategies for more productive and sustainable agriculture requires understanding plant–microbe interactions and the possibility of modulating the plant microbiome [4,5]. Plant growth-promoting bacteria (PGPB) have been extensively studied. 

Currently, most bacterial inoculants used to enhance crop productivity are based on a single strain with a set of plant growth-promoting traits [4,6,7]. Numerous characteristics of PGPB have been identified using in vitro screening assays or inoculation experiments under controlled conditions [8]. However, these characteristics are rarely tested in field conditions and related testing generally neglects the significant aspects of plant–microbe interactions [7].

Another strategy for developing microbial inoculants is to study microbial communities. The information about individual species and possible antagonistic interactions can be used to design synthetic microbial communities to be eventually modified into microbial communities with predictable traits. A key question is how to design bacterial consortia to produce the desired phenotypic outcomes for plants [9]. Not only is it necessary to understand how microbial communities interact and shape the plant microbiome. Moreover, it is of utmost importance to investigate the functions and potential contributions of individual microorganisms at the organismal and molecular levels. This will facilitate the development of manageable and traceable consortia with all the required properties for promoting plant growth [10]. 

Bacteria closely related to the plant rhizosphere are assumed to have a higher potential for interaction and are likely to contribute significantly to the host phenotype. Bacterial communities from the ecological niche of the rhizosphere have probably co-evolved with plants for a long time and share characteristic features such as metabolism, biofilm formation, and others not typical of individuals from other habitats, such as soil, sediments, marine ecosystems, and others [10].

This review focuses on the main approaches to studying the organization of microbial communities of soil bacteria, promoting plant growth and development. Investigating such microbial communities is essential for designing microbial ecosystems with controlled properties for agricultural applications [11,12]. Experimentally determining the forms of microbial interactions in a community remains a significant challenge. Ab initio hypotheses on the interspecies exchange of metabolites can be formulated by analyzing metabolic networks [13,14,15]. The synergistic interaction of bacteria individually exhibiting the properties favorable for plant growth and development at the population level is much more promising than using individual bacteria in agricultural practice [16]. This is due to better rooting, adaptability, and resistance to variable and complex environmental conditions [17,18].

## 2. Agrochemistry and Prospects for Using Plant Growth-Promoting Bacteria

Agrochemicals were once a significant factor in improving crop production. However, research has demonstrated their adverse effects on the sustainability of crop production and environmental safety [19]. Unbalanced use of fertilizers and improper management of crop residues through burning and removal from the field are still practiced in cultivation systems [20,21]. Improper nutrient management strategy resulted in the disturbance of microbial diversity and soil fertility, making it challenging to maintain optimal soil health and plant growth [22,23]. 

The application of PGPB is a promising area for improving soil fertility and stimulating plant productivity in agroecosystems [24]. PGPBs naturally enhance plant growth by the following direct and indirect methods: production of phyto-stimulants such as phytohormones [25] and siderophores [26], stimulation of plant nutrient uptake [27], pest suppression [28,29], and other activities. More importantly, PGPBs can form beneficial associations with plant roots for improved plant growth and resistance to abiotic stresses [30]. The application of PGPB consortia has gained considerable attention, particularly due to the complementary properties exhibited by diverse bacterial strains [31]. Bacterial consortia have proven to be more efficient than a single-species inoculation approach [32]. For example, a consortium of *Bacillus subtilis*, *Bacillus thuringiensis*, and *Bacillus megaterium* was found to significantly enhance metabolic activities, such as an increase in chlorophyll content, riboflavin, L-asparagine, and aspartate accumulation, while also mitigating stress response activity in *Cicer arietinum* under drought conditions [31]. Similarly, a consortium of *Ochrobactrum pseudogrignonense*, *Pseudomonas* sp., and *B. subtilis* was demonstrated to fix nitrogen, solubilize phosphorus, produce indole-3-acetic acid and siderophores, and provide drought tolerance to *Vigna mungo* and *Pisum sativum*, thus promoting seed germination and increasing root and shoot length and chlorophyll content [31].

**Figure 1:**
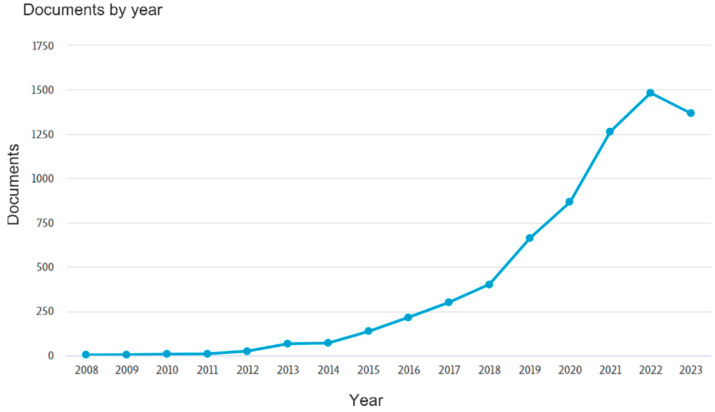
Number of articles indexed by Scopus (1 November 2023) for the “soil microbiome” query from all sources.

The rhizosphere is the narrow boundary zone between plant roots and soil and is apparently one of the most dynamic “boundaries” on Earth [33]. The plant rhizosphere is inhabited by a variety of bacteria, fungi, and protozoa, with numerous interactions occurring within and between kingdoms of living organisms. These interactions can be positive, neutral, or negative and can significantly influence the growth and well-being of plants [1,34]. However, it is only in recent years that scientists have begun to realize the importance of microbe–microbe and microbe–plant interactions. The last decade has witnessed a remarkable growth in research on soil and plant microbiomes, yielding the detailed, albeit mainly descriptive, insights into their extensive taxonomic and functional diversity. According to Scopus, over 5000 articles about the soil microbiome have been published (Figure 1). 

Unfortunately, there is still much to be discovered about the mechanisms that regulate the assembly, diversity, and functioning of the soil microbiome and its beneficial impacts on plants.

## 3. Microbial Consortia

The soil microbiome performs several significant functions at the soil ecosystem level: carbon, nitrogen, phosphorus, and iron cycling and plant growth promotion [35,36]. Central to these functions are the interactions among the species that form soil microbial communities [37]. Individual members of the soil microbiome have enormous combined genomic and metabolic potential. Additionally, novel functions can emerge through community-level metabolic interactions. A better understanding of these interactions can provide a more complete picture of the functional capabilities of microorganisms and communities [38]. 

Captivating data were obtained on the microbial cleavage of plant-derived substances such as cellulose and chitin [39]. The genomic potential for cellulase enzyme production was found in nearly 40% of bacterial genomes. However, only a few microorganisms were demonstrated to digest cellulose in pure culture [40]. Chitinases and cellulases are widely distributed in bacteria [41]. However, their expression is not universal and is controlled differently in different species and at the population level [42]. Chitinases are not connected to cells, suggesting that the breakdown products are available not only to species expressing chitinase but also to other community members. As a result, the community exhibits pronounced interspecific and intraspecific interactions related to the metabolism of chitin and its degradation products.

The native soil microbiome is a complex community of thousands of species with millions of potential interactions between them. Given that no species exists in isolation, it is these interactions that play a pivotal role in determining the characteristics of the soil microbiome. Therefore, for agricultural applications, using multispecies consortia for developing inoculants [43] is considered more promising compared to single-species culture [44,45]. For example, the coexistence of several species within a consortium, with no antagonistic behavior towards each other, can lead to the occupation of a broader range of ecological niches [46], facilitating their colonization of the plant rhizosphere. In addition, a more diverse microbial consortium may possess a more significant number of plant-beneficial functions [47]. As a result, the application of microbial consortia can introduce numerous plant-beneficial functions to the soil. These functions can be concurrently exhibited by different consortium members due to their complementary characteristics.

Inoculated microbes can exert indirect effects through the changes in diversity, composition, and functioning of the rhizosphere microbiome [48,49]. Bacterial inoculation has been shown to cause significant changes in the diversity, composition, and functioning of the soil microbiome, resulting in significant effects, even for subsequent plant generations [50,51]. Such changes can be mediated by microbial competition for resources, potentially altering the balance between dominant and rare taxa [52]. Some inoculated species can interact with the plant to trigger plant-mediated “control” of the microbiome by altering root exudation patterns [53], producing plant-derived antimicrobials [54], or inducing other plant defense mechanisms [55]. Various microbial inoculants can induce relatively large shifts in the functioning of the host plant microbiome [56]. The potential indirect effects of microbial inoculants on the functioning of the rhizosphere microbiome are still poorly understood despite several reports [48,49].

## 4. Microbial Interactions

There are various forms of intermicrobial interactions, such as competition for resources, synergism, and antagonism, to name but a few. These interactions can be altered by the environment [57]. Soil bacteria are capable of distinguishing their microbial competitors and fine-tuning their survival strategies [58]. The secretion of some metabolites by soil microbes has been demonstrated to result directly from interactions with other microorganisms that are in close vicinity [59,60]. To illustrate, it has been discovered that soil bacteria, which do not seem to produce antimicrobials, begin synthesizing specific or broad-spectrum antibiotics when encountering other bacterial species in carbon-limited environments [61]. These findings emphasize the requirement for a comprehensive approach that could surpass the mere study of microbial activity. It is necessary to take into account or mimic the biotic and abiotic conditions of the soil niches commonly inhabited by microbes. 

A hypothesis exists that some microorganisms, referred to as beneficiaries, can “avoid” performing functions in order to optimize their adaptation to the environment. This function loss can be attributed to the presence of “helper” microorganisms performing the same function in the immediate vicinity, providing a stable environment. Hence, soil bacteria may exhibit a metabolic dependence on neighboring microbes. Although it is unknown whether this pattern is typical, it may explain why most soil microbes cannot be cultured individually under laboratory conditions [62]. It is probably for this reason that there is still a lack of physiological, morphological, and ecological characterization for many soil microorganisms due to the failure to isolate, culture, and study pure cultures. There are several strategies of interactions within natural consortia that enable the efficient utilization of resources. These interactions can be categorized following the classical ecological categories of interactions between organisms. The following discussion outlines the primary forms of symbiotic interactions among soil microorganisms.

**Cooperation.** Cooperative interactions are based on functional differentiation and specialization [63], contributing to an increase in overall resource efficiency and a decrease in the formation of byproducts. This type of interaction also allows for more optimal organization of several multi-step processes, such as the degradation of complex organic compounds. Cooperative interactions allocate carbon sources among community members in a non-competitive manner based on metabolic functionality. Cooperation allows parallel processing of the same substrate in different metabolic pathways and is used to create consortia that can simultaneously ferment pentose and hexose sugars, which is often unattainable in monocultures because of catabolic repression [64].

**Commensalism.** Another standard bacterial interaction mode is commensalism. This is when the activities of one community member provide an ecological niche for others with no benefit or cost to itself. One example of commensalism is metabolite exchange whereby a producer organism excretes byproducts with no benefit or cost to itself but provides metabolites to other members of the community [65].

**Mutualism.** Mutualism is a common natural phenomenon defined as a relationship that is beneficial to all participants. For example, a community member consuming organic acid removes inhibitory byproducts from a producer population [66]. Mutualistic interactions can be facultative or obligatory. In obligatory mutualistic interactions, organisms fail to perform a particular action when separated from the interaction partner. One example of such interactions is the syntrophy of secondary-fermenting bacteria with hydrogen-consuming microorganisms or methanotrophic Archaea with sulfate-reducing bacteria [67].

Optional mutualistic interactions are beneficial to both interaction partners but are optional in pure culture. One example is the coaggregation of bacteria that can exist in pure culture but undergo specific surface interactions when incubated together under appropriate conditions. This type of cellular contact can promote metabolic interactions between these bacteria [68].

Competition and parasitism are examples of antagonistic, antibiosis microbial interactions.

**Competition.** Competitive interactions, as well as predator–prey or parasite–prey interactions, are beneficial to only one interaction partner. Competition for nutrients is the most frequent antagonistic interaction in the microbial world [69]. In this case, microorganisms try to utilize nutrients while gaining advantages over other microorganisms. Microorganisms have developed several strategies for successful competition, such as biosynthesis and secretion of antibiotics, close association of enzymes of metabolic pathways, and uptake of degradation products.

**Parasitism.** A parasitic interaction is established if the recipient forces the producer to release an intermediate compound to be used by the recipient. One example is the parasitic growth of *Pseudomonas aeruginosa* in co-culture with the chitinolytic bacterium *Aeromonas hydrophila* [70]. *P. aeruginosa* uses secondary metabolites to control metabolism in such a way that chitin is not completely oxidized to acetate, which serves as a substrate for *Pseudomonas aeruginosa* growth.

## 5. Modeling Consortia

**Figure 2:**
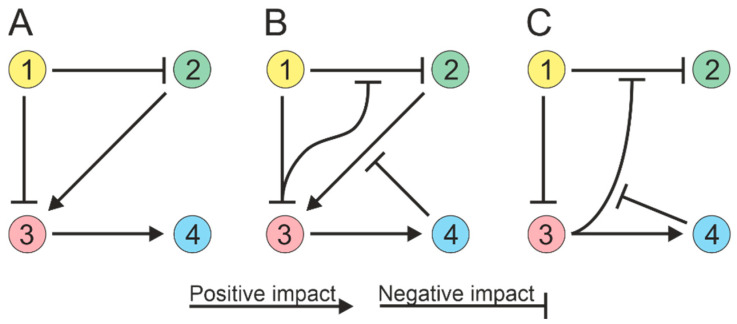
Soil microorganisms can exhibit high-order interactions, with interactions between two species being influenced by other species (1 to 4). (**A**) Paired interactions in a community; species directly influence each other. (**B**) Three-way community interactions; one species can modulate the interaction between two others. (**C**) Quadripartite community interactions.

The process of modeling ecological interactions assumes pairwise interactions (Figure 2A). However, soil microorganisms can interact in higher-order combinations, i.e., interactions between two species can be regulated by other species [71,72]. For example, a microbial species can produce an antibiotic that inhibits the growth of competing species, and this inhibitory effect can be attenuated by a third species that produces an enzyme that cleaves the antibiotic [73,74]. Thus, the third species can alter the interactions between the antibiotic-producing strain and susceptible species without directly affecting either of them individually (Figure 2B). The activity of the enzyme responsible for antibiotic degradation can be impeded by compounds generated by the fourth species [75], resulting in a four-way interaction (Figure 2C). Thus, possible strategies for interspecific interactions must be considered when creating microbial communities.

**Figure 3:**
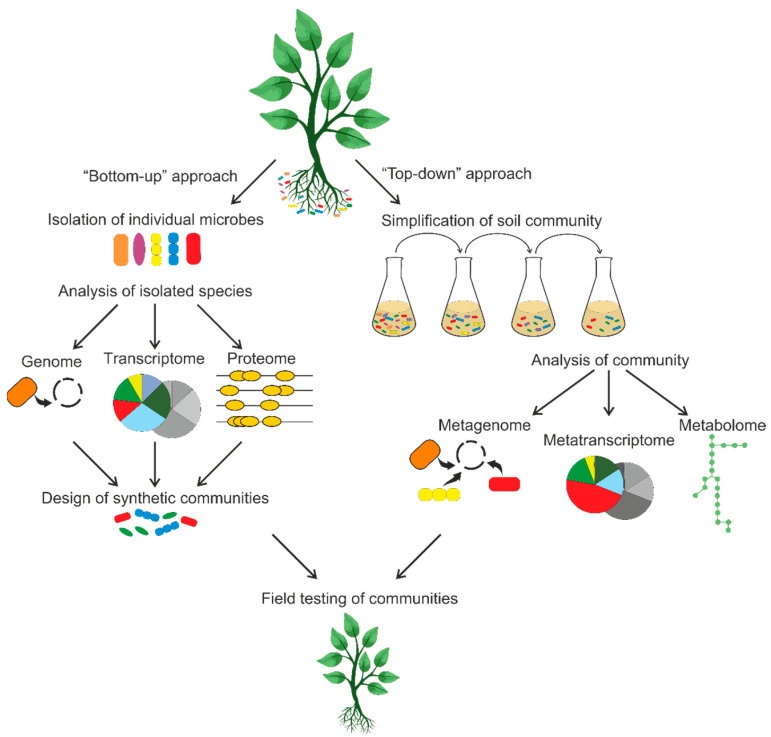
Schematic representation of two approaches to studying microbial communities. The “bottom-up” approach involves isolating and studying individual species and then creating synthetic consortia. The “top-down” approach involves simplifying the original soil community and then characterizing it using omics techniques.

Given the multitude of biochemical reactions conducted by the soil microbiome, researchers aim to establish model soil consortia to comprehensively investigate the metabolic interactions among species. Synthetic microbial communities are simplified ones. Ideally, a model consortium should comprise a manageable and reproducible number of species, allowing the population dynamics and specific metabolic and signaling interactions among its members to be experimentally analyzed [76,77]. The knowledge acquired from the analysis of these communities of reduced complexity will help to reveal details of metabolic pathways and interspecific interactions that occur in natural soil communities. Next, we shall consider two approaches to studying consortia that are summarized in Figure 3. 

### 5.1. The “Bottom-Up” Approach

The “bottom-up” approach for analyzing the soil microbiome is to combine individual strains to form “synthetic” consortia [75,78]. There are a few drawbacks to be considered. It is crucial to know which species interact in the natural environment because there is a risk of combining species that do not interact in the natural habitat, which would lead to the obtainment of results that would be difficult to operationalize. Another disadvantage of this approach is the likelihood of establishing consortia with a limited number of members, indicating that only a fraction of the interacting soil components can be taken into account. Investigating synthetic communities has several advantages, and the simplicity of the analysis can be useful in unraveling particularly complex interactions. Creating a microbial system from one or more microorganisms allows one to construct a physical model with higher reproducibility, lower variability, and a lower number of unknowns than in the original system. Analyzing a simplified consortium allows one to study the contribution of individual species to the overall metabolism of the community. Pure cultures in co-culture are used to describe model bacterial consortia, making it possible to determine the role of individual species in this consortium.

The first step is to narrow down the most important components of the microbial community and, as a consequence, their functions. As a result, one can identify and select the members of the microbial community that are crucial for the process of research interest, thereby significantly reducing the complexity of the system under study [79]. The second step is to analyze the ecologically relevant function of most soil microorganisms. Thus, soil microorganisms can be isolated, and those capable of performing different functions (e.g., nitrogen fixation, phosphate solubilization, siderophore synthesis, and auxin production) can be identified. In the future, this information can be used for more accurate mathematical modeling of processes in the consortium.

Strains selected for creating consortia are tested for in vitro compatibility [80]. For this purpose, bacterial inoculum is applied to a nutrient agar dish coated with the strain under test. The plates are stored at 28 ± 2 °C and monitored for up to 72 h. The absence of a zone of inhibition around the bacterial colony indicates that the bacteria are compatible with each other [81].

Most synthetic microbial consortia are created using pairwise interactions [82,83]. Increasing the diversity of a microbial community also increases its complexity [84]. It is the complexity of analyzing these interactions that makes it difficult to construct and predict dynamics, even for small communities [85].

### 5.2. The “Top-Down” Approach

A “top-down” approach for investigating the diversity and complexity of the soil microbiome is to create low-complexity consortia [86]. This method utilizes dilution and cultivation on complex carbon sources (e.g., N-acetylglucosamine and chitin). Dilution promotes a reduction in species richness of the community, and a stabilization of the consortia (after about 3–5 weeks of incubation) yields several microbial communities. However, the resulting consortia are still comparatively complex and contain up to several hundred species [87]. By adopting such an approach, the community can independently shape the species composition of a consortium and enhance its growth through necessary interactions. It should be noted that the type of carbon source has an impact on the rate and direction of consortium development [88]. The “top-down” approach involves identifying the community function to understand the community structure and dynamics. The valuable availability of a particular “function” for a microbial community allows one to measure the degree and stability of that function and, thus, to compare different communities with the same function [89].

The “top-down” approach outperforms a “bottom-up” approach in generating representative communities [90]. Moreover, the larger community allows more soil functional potential and species interactions to be captured and studied. For example, applying the “top-down” approach resulted in 35 species being detected, with approximately 13 being the dominant ones compared to 2–3 dominant ones detected using the “bottom-up” approach [87]. A significant attribute of this approach is that the community created represents, to some extent, the natural soil microbiome and hosts species from a variety of taxa, from families to genera. 

The “top-down” approach proves useful for studying relationships between key community properties, such as diversity, function, and stability, while also providing a mechanistic understanding of community structure [89]. It is not unlikely that difficult-to-cultivate species can be studied exclusively using this approach due to their need for interaction with other members and their inability to exist independently without additional biochemical information [76].

It is worth noting that employing the two approaches considered above has resulted in valuable discoveries in the plant microbiome [88]. Combining the strengths of both approaches to study one model community can be highly effective. The “top-down” approach can reduce the soil microbiome to a more manageable size and identify interacting members. This can be followed by pairwise comparison (simple “bottom-up” approach) and a more detailed description of their interactions. Next, we shall discuss some cases of obtaining synthetic microbial consortia. 

### 5.3. Practical Applications of Synthetic/Artificial Microbial Communities

Synthetic consortia are small consortia of microorganisms designed to mimic and analyze the microbiome function and structure in vivo. Such consortia represent a new frontier for synthetic biology as they allow more complex problems to be solved compared to monocultures. 

The purpose of creating synthetic consortia is to reduce the complexity of the natural microbial community while preserving some of the original interactions between microbes and hosts, providing a repertoire of functions that cannot be achieved by a single microorganism [91,92,93]. Creating synthetic microbial communities in the laboratory involves selecting certain microorganisms according to their ability to stimulate plant growth, protect plants from pathogen infestation, or provide nutrition to plants. Subsequently, microbes exhibiting different characteristics are amalgamated into synthetic consortia, leading to enhanced community stability through synergistic interactions among the constituents [94].

It has been suggested that microbial communities with increased metabolic activity should be modeled by selecting species that are not functionally too close or too distant [95]. However, the universality of this hypothesis has yet to be confirmed experimentally. Strain selection is assumed to be based, among other things, on functional genes rather than on taxonomic classification [96].

Several studies have succeeded in demonstrating that engineered synthetic consortia stimulate plant growth and disease resistance. For example, two synthetic consortia with different bacterial genera have been developed to stimulate tomato growth or suppress symptoms of tomato fusarium wilt [97]. Several other studies have also reported positive effects of microbial consortia [94]. A consortium of four species of bacteria *Stenotrophomonas rhizophila*, *Xanthomonas retroflexus*, *Microbacterium oxydans*, and *Paenibacillus amylolyticus* has been shown to enhance drought tolerance of *Arabidopsis* plants [98].

Three consortia were developed based on paired and triple interactions of phosphate-solubilizing bacteria for wheat inoculation, with the first consortium comprising *Enterobacter* sp. and *Ochrobactrum* sp., the second consortium comprising *Pantoea* sp., *Enterobacter* sp., and *Ochrobactrum* sp., and the third consortium comprising *Ochrobactrum* sp., *Pseudomonas* sp., and *Bacillus* sp. Rhizosphere analysis revealed a significant increase in linear root growth after inoculation with these consortia. Inoculating wheat grown under natural and greenhouse conditions resulted in a positive correlation between enhanced plant growth and increased soil phosphorus content [84].

A consortium of two rhizobacteria *Agrobacterium sp.* and *Kitasatospora* sp. and the arbuscular mycorrhizal fungus *Claroideoglomus* sp. was developed. The application of these rhizosphere bacteria in the form of consortia was shown to stimulate the growth of teak tree Tectona grandis more effectively than individual application and to contribute to a significant increase in seedling biomass [99]. There are publications reporting consortia containing more than two microorganisms, e.g., *Xanthomonas* sp., *Stenotrophomonas* sp., and *Microbacterium* sp. [100]; *Brevibacillus fluminis*, *B. agri*, and *Bacillus paralicheniformis* [101]; *Bacillus cereus*, *B. subtilis*, and *Serratia* sp. [102]; *Ochrobactrum pseudogrignonense*, *Pseudomonas* sp., and *Bacillus subtilis* [103].

A key factor in developing a microbial consortium, which is essential for the successful functioning of the included microorganisms, is the compatibility of the individual microorganisms. Bacteria of the genera Bacillus and Trichoderma were found to perform well both in consortia and in individual inoculation, and Pseudomonas was reported to perform better in synthetic consortia than when inoculated individually [104]. These properties encountered when creating consortia are likely to be the clue to utilizing PGPB for agroecosystems. The potential contribution of individual microorganisms can be assessed by altering the composition of a synthetic community through the addition, removal, or replacement of microorganisms [10]. For example, removing a single strain of *Enterobacter cloacae* in maize destroyed the functionality of the microbial community, which reduced the severity of phytophthorosis disease [92].

Despite the apparent advantages in terms of practical use, designing consortia for field applications has proven to be much more complicated than when dealing with individual strains. Most attempts to grow bacteria together as part of artificial microbial communities have failed [105,106], accounting for the actuality of microbial compatibility. For example, communities that incorporate bacteria with pronounced antagonistic interactions exhibit unstable coexistence of microorganisms, which eventually leads to the dominance of one or more species [107]. Combining compatible microorganisms with complementary actions that are effective under different conditions will allow more versatile consortia to be developed in the future. It is imperative that the consistency of action of individual strains be tested and validated in field trials in different geographical regions on different crops in order to produce commercially successful products. That seems to be a key step for the successful and widespread application of this highly promising sustainable farming technology.

### 5.4. Identification of Microbes with Key Characteristics

Selecting microbes with important properties for agricultural applications mainly involves in vitro screening and sampling of well-known taxa or activities favorable to plant growth and development, such as atmospheric nitrogen fixation, phosphorus solubilization, phytohormone, and siderophore production [26,27]. Unfortunately, inoculating plants with preparations of microorganisms obtained using these traditional approaches often fails to result in long-term stable interactions with plants under field conditions and subsequently in satisfactory results [108,109]. For a strain to be considered successful, it must effectively compete with existing microorganisms in the soil, establish a strong presence in the plant root system, and form stable and sustainable associations, even in the face of potential changes in the environment and soil microbial composition throughout the growing season. 

One strategy to overcome the challenges is to select microorganisms based on the diversity of plant microbiota. The analysis of 16S rRNA gene sequencing data has revealed that certain groups of soil bacteria can successfully colonize plant roots and establish and maintain permanent relationships with them, whatever the environmental changes or the plant developmental stages [110,111]. Incorporating members of dominant groups referred to as the “core microbiome” into synthetic communities can mitigate the potential inefficiencies observed when strains are outcompeted by the natural soil microbiota. Using the keystone groups of microorganisms can be highly effective in colonizing the plant rhizosphere [92]. For example, bacteria colonizing sugarcane roots have the potential to colonize the maize root system as well as promote its linear growth [112]. Genome sequences of core microbiome members isolated from sugarcane indicate that compared to non-core microbiome strains, resistant colonizing strains are enriched in carbon metabolism genes [7].

The growing number of reference genomes and metagenomes in publicly available databases is an important basis for identifying soil bacteria with desirable characteristics beneficial to plant growth and development. Using bacterial genome sequencing data and gene expression profiles allows for a more efficient selection of microorganisms with functional traits or metabolic characteristics useful for plants [113,114]. In the future, this knowledge will contribute to the development of optimal combinations of soil bacteria to create inoculant consortia.

### 5.5. Development and Stabilization of Microbial Communities

Synthetic microbial communities can be stabilized in the short term, up to 36 h, and in the long term, up to two weeks [110,115]. Given these data, consortia need to be given enough time to stabilize, e.g., several weeks, especially when using a “top-down” approach, before they can be maintained and revived.

Significant changes in the relative abundance of some taxa are observed during the first week of incubation in soil, followed by stabilization after two weeks [87]. Stabilizing the soil microorganism community may require 3 to 5 weeks, with stability observed for several months afterward [86]. Thus, when studying model consortia, it is worth considering the period required to achieve stability after inoculating the consortium into new soil.

## 6. Designing PGPB Consortia That Can Reduce the Response to Abiotic Stress

**Figure 4:**
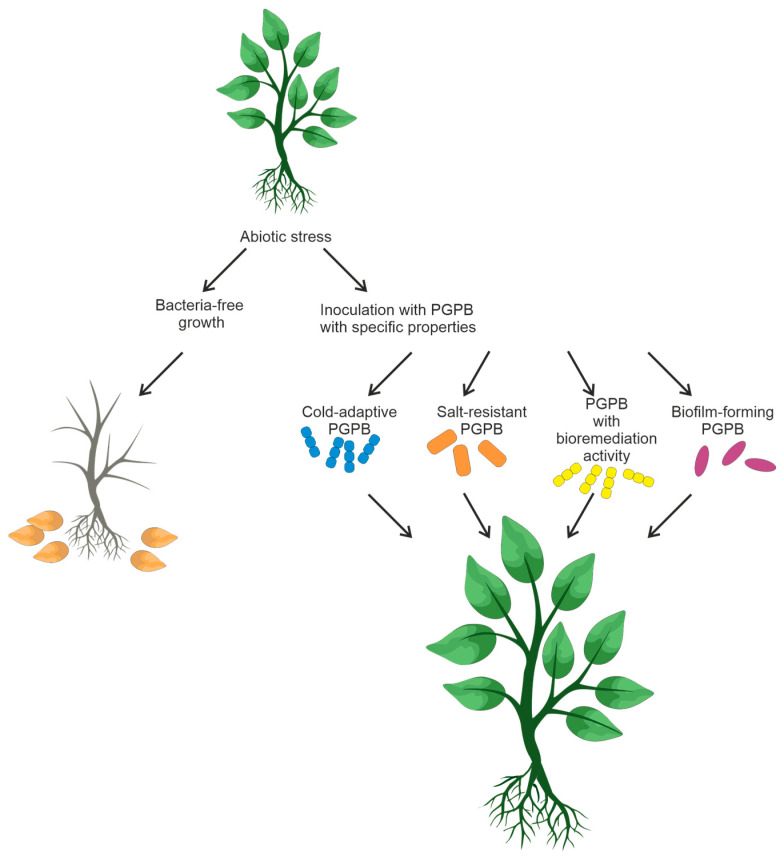
Inoculation of plants with soil PGPB capable of reducing abiotic stress.

The design and creation of bacterial consortia is a non-trivial task that demands careful consideration. It is crucial to ensure the absence of antagonistic interactions among the members of the bacterial mixture to enable their coexistence. Moreover, bacteria selected for bacterial mixtures should be capable of enhancing plant growth, bioremediation, and tolerance of unfavorable conditions in crop fields. Figure 4 summarizes the information on soil bacteria and their possible consortia that could favorably affect plant growth.

Food crop productivity is affected by various abiotic factors, such as salinity, drought, and temperature [111,116]. Some functional features of rhizobacteria (biofilm formation, bioremediation, resistance to soil salinity, and low temperatures) have an impact on plant survival under unfavorable conditions [117,118]. PGPR-based microbial strategies can be efficient in overcoming the negative effects of abiotic factors. Some bacteria are capable of forming a biofilm that can enhance resistance to antibiotics, heat, UV radiation, and other environmental stresses [119,120]. When exposed to unfavorable conditions, soil bacteria secrete exopolysaccharides that form an organomineral envelope, also known as biofilm. These polysaccharides consist of complex mixtures of high-molecular-weight polymers (MW ≥ 10,000) [121]. For example, wild-type *B. subtilis* strains have been demonstrated to form a robust biofilm, to successfully colonize on the surface of tomato roots, and to be highly efficient in biocontrol against the pathogen *Ralstonia solanacearum* [122].

It is common for bacteria to produce exopolysaccharides under conditions of heavy metal stress and high salinity [123]. However, high-quality exopolysaccharides can only be produced by halo- or drought-tolerant rhizobacteria that can tolerate and survive under harsh conditions [124].

Studies of the exopolysaccharides of soil bacteria using confocal laser scanning microscopy combined with lectin binding techniques have proven invaluable for biofilm characterization [125]. However, detecting bacterial exopolysaccharides in soil is extremely difficult due to (i) physical obstruction by mineral particles, (ii) challenge of sample preparation, and (iii) typical content of humic compounds and degradable plant carbohydrates that may interfere with the binding of the fluorescent label [126].

Soil salinity is regarded as the most significant among abiotic factors [127]. Experiments on inoculation of salt tolerant PGPB have demonstrated remarkable results in increasing agricultural productivity of saline soils [128,129]. The mechanisms contributing to plant growth stimulation have already been reviewed [26,27]. Aside from the mechanisms inherent in PGPB, salt-resistant bacteria exhibit specialized mechanisms essential for salt stress tolerance and plant growth promotion. For more details, the reader is referred to the review by M. Numan et al. [130]. 

In recent decades, heavy metals have become a major environmental pollutant [131,132]. Several PGPB strains have been demonstrated to be tolerant to heavy metals even at high concentrations [133,134]. The metal tolerance of numerous halotolerant and moderately halophilic bacteria is due to their superior sorption capacity and numerous potential active chemisorption sites on the cell wall [135]. Notably, these bacteria reduce the adverse effects of heavy metals on plant health and allow plants to tolerate high metal concentrations.

Temperature is another crucial factor affecting plant growth. Low temperature puts plants under stress and affects microbial growth and activity. Agricultural cultivation in cold regions requires biofertilizers that can work at low temperatures. Inoculation of cold-adapted microbial inoculants PGPB significantly promotes plant growth under low-temperature conditions [136]. Cold-adapted PGPR, such as *Burkholderia* and *Pseudomonas* spp., have been described [137,138]. Additionally, bacteria from soils of the northwestern Indian Himalayas have been described [138]. Bacillus strains isolated from the rhizosphere of local potato varieties from the Andean highlands have been demonstrated to act antagonistically in vitro against plant pathogens [139] and can promote plant growth [140].

## 7. Multi-Omics Approaches for Studying Microorganisms and Their Consortia

Traditional microbial culturing methods and modern methods such as pyrosequencing or NGS [141,142] are used to characterize and compare microbial communities and identify individual functions in different ecological niches. These methods have specific advantages and limitations that should be taken into account when designing consortia. It is essential to isolate pure cultures of bacteria in order to study them in detail, identify specific traits, and directly determine the genetic components that underlie useful phenotypes [143,144]. However, only some soil microorganisms can be cultured in laboratory conditions. Metagenomic analysis approaches and omics technologies allow the structure of the microbial community and the functions of its individual components to be determined [145,146]. “Collective phenotypes” of interacting species within the soil microbiome are referred to as the metaphenome [147]. However, the gigantic diversity of the soil microbiome [148], with thousands of species and billions of potential interactions between species [148,149], complicates the analysis of the specific interactions underlying the soil metagenome.

Complete metagenomic sequencing provides information on the genomes of all microorganisms and allows the functional and metabolic potential of a community to be characterized [150]. However, not all genes are expressed at any given time, and the total DNA extracted from soil may contain sequences from currently inactive populations. Nevertheless, high-throughput approaches have identified functional signatures of some rhizosphere and endosphere communities [142,151], and it is this approach that can answer the question of which microbial genes are enriched in a particular microhabitat.

The soil metatranscriptomics is used to determine the functions performed by individual members of the soil microbiome [152,153]. Metaproteogenomic approaches enable the exploration of active functions and metabolic pathways [154]. These two approaches provide insight into the timing and location of specific gene expression. However, such studies are usually based on relatively shallow metatranscriptomes with read depths insufficient to cover all the members of a community; thus, these studies investigate only the most abundant and transcriptionally active species and genes. Similarly, despite the advances in soil metaproteomics [155,156], the dynamic range and depth of coverage are still limited to “surface” analysis of the soil microbiome.

Metaproteomic measurements of microbial communities do provide reliable and reasonably accurate estimates of microbial population sizes [157] because proteins make up to 40–60% of bacterial cell biomass [158] and have a linear correlation with cell mass and volume [159]. Estimates of relative cell population sizes in the organism community, based on the summation of protein abundance or content, suggest that the habitat has achieved population equilibrium and stabilization. Metaproteomics allows large-scale identification and quantification of microbial community proteins, facilitating the characterization of microbial identity, functional roles, and interspecific interactions in the community [160]. Additional analysis is also performed for extracellular fractions, typically proteins secreted by cells. Additionally, characteristics of the extracellular fraction may include the abundance of proteins produced by cell lysis, allowing recent microbial death to be detected [57].

Recent advances in optimizing bioinformatic pipelines for metaproteomic analysis using de novo peptide sequencing have also made it easier to identify and characterize post-translational modifications in proteins, thus facilitating the analysis of microbial regulation and adaptation [161]. Post-translational modifications of proteins are an important but still poorly understood mechanism used by microorganisms to rapidly respond to changing conditions [162,163]. Among the biologically relevant post-translational modifications in the proteomes of bacterial isolates, the most common are methylation, dehydration, oxidation, and hydroxylation [164,165]. The effects produced by these types of modifications on proteins can be quite diverse, but they are often related specifically to the changes in protein–protein interactions [166,167].

To summarize, although multidomain approaches do hold promise as technologies for assessing the functions performed by interacting members of the basic soil microbiome, interpreting specific interspecies interactions is still a challenging task to be solved in the future.

## 8. Analysis of Metabolic Networks of Microbial Communities

Reconstructing metabolic networks of microbial communities is a challenging task. Even when dealing with a single species, the iterative process of genome-wide reconstruction of the metabolic network demands a substantial amount of time [168]. Modeling a community is a more challenging process due to the increased complexity associated with interactions between species. The traditional practice of designing community metabolic networks focuses on reconstructing high-quality individual networks so that their combination may provide quantitative predictions of metabolic interactions and community behavior [169]. However, in practice, such an approach does not take into account possible microbial interactions. The minimum information required includes the genome sequence determining the key metabolic functions and physiological data, such as growth conditions for more accurate modeling of networks. In general, the more physiological, biochemical, and genetic information available for the organism under study, the better the predictive ability of the resulting models. This is evident, given that the network evaluation and validation process relies on comparing predicted phenotypes with experimental observations [170,171].

Current sequencing methods fail to read the entire genome at a time. Therefore, all sequencing protocols start by cutting DNA into smaller fragments that the sequencer can read [172]. The sequences of overlapping fragments resulting from sequencing are called contigs. If the sequencing coverage is deep enough, contigs can be assembled into one or more scaffolds covering the complete genome. Subsequently, it is essential to ascertain the location of the genes and comprehend their respective functions. The most important genes for metabolic reconstruction are those that function as enzymes and transport proteins. Let us consider the protocol of the reconstruction process in terms of metabolic networks. The same approach can and has been implemented to reconstruct signal transduction [173,174] and transcription and translation networks [175]. The process of metabolic network reconstruction involves four main stages: creating a sketch of the reconstruction, manually refining the reconstruction, converting the reconstruction into a mathematical model, and debugging. The stages from the second to the fourth are repeated until the model predictions are similar to the phenotypic characteristics of the target organism and/or all experimental data for comparison have been exhausted. For the details of all steps of metabolic network reconstruction, the reader is referred to the work of Thiele I. and Palsson B. [168].

### Strategies for Design of Microbial Community Networks

Community networks can be constructed from the genomes of individual species. Figure 4 illustrates three alternative approaches to constructing the microbial community metabolic models described in this section.

**Figure 5:**
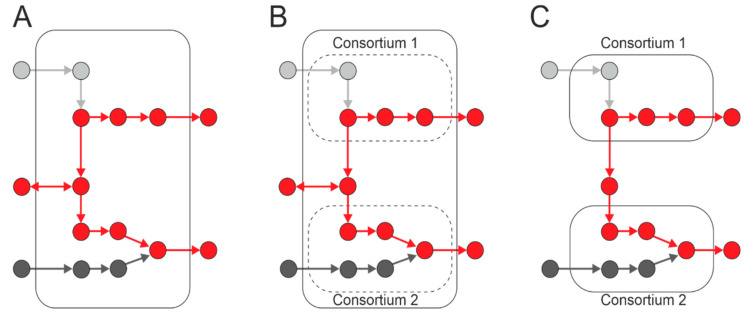
Strategies for constructing community metabolic models for a binary consortium: (**A**) mixed modeling approach, (**B**) partitioned network modeling, (**C**) dynamic multispecies modeling. The metabolic pathway common to both consortia is indicated in red. These schemes are presented according to [178] with some modifications.

The simplest level involves a mixed approach and treats the microbial community as a single superorganism [176,177] (Figure 5A). Metabolic pathways and transmembrane transport components of all community members are combined, with species boundaries ignored. The main application of the mixed approach is to analyze the interactions between the community and the environment. Constructing a mixed network requires complete genome sequences of all community members or in-depth metagenomic analysis. However, it does not necessitate the studies at the level of individual species. 

Predicting interspecific metabolic interactions requires modeling at the level of different species (Figure 5B). This can be achieved by treating gene networks of individual species as community compartments with structures analogous to those of eukaryotic metabolic networks, with inter- and intra-compartmental activity assumed to be in a quasi-stationary state. Dynamic interactions between community members can be investigated by overlaying kinetic expressions at the species level. Multispecies metabolic modeling begins with designing metabolic models for the individual species that make up the community. 

The formation of a community derived from a natural environment necessitates the implementation of a species-level metabolic model-building process. This process involves analyzing metagenomic data, partitioning the collected contigs into species-level cells, annotating genes, and constructing a model based on the available data (Figure 5C).

The next step is to evaluate the ability of the constructed models to accurately predict which pathways will be active in the microorganisms when they form a consortium. For this purpose, RNAseq data of the consortium under study are collected, and reads are mapped to individual genes. Each gene is categorized as “active” or “inactive” depending on its expression level. Finally, transcriptome analysis [179] is performed to reconcile the predictions of the metabolic network with gene activity calculated from RNA sequencing data. As a result, reactions associated with inactive genes are excluded from the constructed network.

## 9. Economic Impact of PGPB Application

Fortune Business Insights has estimated the agricultural market for microbial fertilizers to be over USD 5 billion in 2021 and it is expected to grow to nearly USD 16 billion by 2029. Bacterial fertilizers account for more than 75%, with liquid fertilizers holding approximately 60% [180]. Fertilizers containing nitrogen-fixing bacteria account for about 80% [181], while fertilizers with phosphate-solubilizing bacteria make up about 15% [182]. PGPB-based biofertilizers are produced and approved for use in many countries, including China, India, the European Union, USA, Argentina, Australia, Brazil, Canada, Colombia, Cuba, Egypt, Japan, Kenya, Malawi, Nigeria, Russia, Spain, Sri Lanka, South Africa, UK, Uruguay, and Vietnam. The species and genera of bacteria used in commercial biofertilizer formulations are summarized in Table 1.

Table 1 demonstrates the utilization of various bacterial species in multiple commercial biofertilizers. The fact that different bacterial strains can vary significantly is widely recognized, leading to a lack of evidence supporting multiple plant growth-promoting activities in the bacterial strains used in these biofertilizers. However, the literature data indicate that such “multifunctional” (e.g., containing both phosphate solubilization and nitrogen fixation genes and exhibiting these activities at least in vitro) bacteria do exist in nature at least [183]. A number of biofertilizers are already produced using PGPB consortia, and we believe that creating biofertilizer preparations containing consortia and “multifunctional” bacteria is the future of agricultural biofertilizers.

## 10. Conclusions

Modern agricultural practices commonly make use of inoculants consisting of a single strain isolated through in vitro screening of plant growth stimulation activity or inoculation experiments under controlled conditions. Although these strategies are widely used, they neglect important aspects of plant–microbe interactions. Due to the highly diverse and complex nature of the plant rhizosphere microbiome, which is sustained through extensive interactions between microbes and their hosts, a more comprehensive understanding can only be achieved by implementing sophisticated research methodologies. 

In recent years, the studies of the plant rhizosphere microbiome have provided new insights into microbial diversity, abundance, distribution, dynamics, and functions. The emergence of various microbiome-related phenotypes is attributed not to the impact of a single species but to the cooperative interaction of multiple species that effectively execute a common function. Microbial interaction networks in soil are often analyzed to detect the co-occurrence among community members and to identify the key taxa. These taxa may be critical to microbial communities, and their removal can cause dramatic shifts in microbial community structure and function. Given the complexity and context-specific nature of ecological interactions among microorganisms, which involve both structural and random interactions, it is often difficult to discern the contributions of different members within a microbial consortium to a particular function or phenotype.

Incorporating microorganisms to facilitate plant growth and development in agriculture is increasingly regarded as a promising alternative for enhancing crop productivity in sustainable agriculture. However, developing stable and efficient consortia for agriculture requires using approaches that take into account recent advances in soil microbiome research, such as multi-omics analysis. In this case, the combination of different approaches for microbial community research is expected to create new opportunities for developing sustainable agricultural production.

## Figures and Tables

**Table 1:** PGPB used as commercial biofertilizers in agriculture [181,182].

Type of Bacterial Biofertilizer	Species (Genus) of Bacteria
Nitrogen fixation	*Acetobacter diazotrophicus*, *Acetobacter* sp., *Azoarcus* sp., *Azospirillum brasilense*, *Azospirillum lipoferum*, *Azospirillum* sp., *Azotobacter chroococcum* ^2^, *Azotobacter vinelandii*, *Azotobacter* sp., *Bacllius megaterium* ^1^, *Bacillus subtilis* ^1,4^, *Bradyrhizobium elkanii*, *Bradyrhizobium japonicum*, *Delftia acidovorans*, *Mesorhizobium ciceri*, *Penicillium bilaii*, *Paenibacillus polymyxa*, *Rhizobium japonicum*, *Rhizobium* sp., *Rhizophagus irregularis* ^1^
Phosphate solubilization	*Bacillus megaterium* ^1^, *Bacillus mucilaginosus*, *Bacillus polymyxa*, *Bacillus subtilis* ^1^, *Bacillus* spp., *Frateuria aurantia*, *Glomus intraradices*, *Pseudomonas fluorescens* ^3^, *Pseudomonas striata*, *Rhizophagus irregularis* ^1^
Solubilization of Potassium and Zinc	*Frateuria aurantia*, *Thiobacillus thiooxidans*
Stimulation of plant growth	*Azotobacter chroococcum* ^2^, *Pseudomonas azotoformans*, *Pseudomonas fluorescens* ^3^
Biocontrolling	*Bacillus subtilis* ^4^, *Brevibacillus laterosporus*, *Paenibacillus chitinolyticus*, *Pseudomonas chlororaphis*

^1^ Bacteria possessing both nitrogen fixation and phosphate solubilization activity. ^2^ Bacteria possessing both nitrogen fixation phytostimylator activity. ^3^ Bacteria possessing both phosphate solubilization and phytostimylator activity. ^4^ Bacteria possessing both nitrogen fixation and biocontrolling activity.
